# Establishment and Validation of an Orthotopic Metastatic Mouse Model of Colorectal Cancer

**DOI:** 10.1155/2013/206875

**Published:** 2013-04-21

**Authors:** Ashwani Rajput, Ekta Agarwal, Premila Leiphrakpam, Michael G. Brattain, Sanjib Chowdhury

**Affiliations:** ^1^Division of Surgical Oncology, Department of Surgery, MSC 07 4025, University of New Mexico Health Sciences Center, 1 University of New Mexico, Albuquerque, NM 87131-0001, USA; ^2^Eppley Cancer Center, University of Nebraska Medical Center, 985950 Nebraska Medical Center, Omaha, NE 68198-5950, USA

## Abstract

Metastases are largely responsible for cancer deaths in solid tumors due to the lack of effective therapies against disseminated disease, and there is an urgent need to fill this gap. This study demonstrates an orthotopic colorectal cancer (CRC) mouse model system to develop spontaneous metastasis *in vivo* and compare its reproducibility against human CRC. IGF1R-dependent GEO human CRC cells were used to study metastatic colonization using orthotopic transplantation procedures and demonstrated robust liver metastasis. Cell proliferation assays were performed both in the orthotopic primary colon and liver metastatic tumors, and human CRC patient's specimen and similar patterns in H&E and Ki67 staining were observed between the orthotopically generated primary and liver metastatic tumors and human CRC specimens. Microarray analysis was performed to generate gene signatures, compared with deposited human CRC gene expression data sets, analyzed by Oncomine, and revealed similarity in gene signatures with increased aggressive markers expression associated with CRC in orthotopically generated liver metastasis. Thus, we have developed an orthotopic mouse model that reproduces human CRC metastasis. This model system can be effective in developing new therapeutic strategies against disseminated disease and could be implemented for identifying genes that regulate the development and/or maintenance of established metastasis.

## 1. Introduction

Colorectal cancer (CRC) is a major cause of cancer-related deaths in the United States [1]. The high mortality rate in CRC as well as other solid tumors stems out mainly from the metastatic dissemination of cancer cells to distant organ sites [1, 2]. Metastasis is a complex, multistep process that is presently under intense study [3]. The process of metastasis requires cancer cells originating from the primary tumor to overcome several layers of barrier to initiate secondary tumor deposits at a distant site which are often characterized by highly aggressive phenotypes [3, 4]. There is considerable heterogeneity in the occurrence of metastasis based on the type of cancer cell. Certain subtypes of disseminating breast cancer cells which have demonstrated the ability to survive and colonize at distant organ sites are usually restricted to a small population of tumor-initiating cells [3, 5]. In contrast, relatively large populations of lung adenocarcinoma cells are able to survive the multistep metastatic process and frequently form aggressive secondary lesions [2, 3]. Talmadge and colleagues [6] have posited that the primary and metastatic phenotypes observed in different cancer cells are a consequence of specific cellular properties that are dependent on both the cancer cell's intrinsic characteristics and its interactions with the host environment, which differs extensively between tissues and organs. However, the molecular mechanisms involved in the multistep dissemination process are not completely elucidated. 

Numerous *in vitro *techniques have been utilized to characterize the fundamental processes integral to the metastatic cascade, including cancer cell motility, invasion, and growth [3]. These *in vitro* model systems including fluorescent and/or bioluminescent reporter molecules have successfully been utilized to underpin metastatic steps in single-cell or cell-cluster levels [3, 7, 8]. However, such studies can only allow for dissection of certain early steps of metastasis in isolation due to lack of the intrinsic properties and complexity associated with the metastatic process in specific tissue context [3]. In recent years, the study of cancer progression and metastasis *in vivo* has been evolved significantly around two general strategies in mice models: genetically engineered cancer models (referred to here as GECMs) and spontaneous transplantable cancer models (referred to here as STCMs) [9–14]. The GECMs are driven by tissue-specific genetic mutations of different oncogenes that generate reproducible information on tumor initiation and progression enabling the study of early steps in the metastatic process [9–13]. Limitations of the GECMs are its low metastatic rates and restricted dissemination to the lymph nodes or lungs. Various STCMs have been developed either in syngeneic or xenograft models to study the late stage metastatic process like metastatic colonization of distant organ sites that involves the engrafting of human or mouse tumor tissues into mouse hosts [14]. Syngeneic models allow for the study of tumor microenvironment but are restricted to the study of mouse cancer cell metastasis [3]. To date, xenograft STCMs are the model of choice for the study of metastatic colonization of human cancer cells *in vivo* [3]. 

In this study, we have utilized the IGF1R-dependent GEO human CRC cell line [15, 16] to study CRC metastasis using an orthotopic metastatic mouse model system that utilized transplantation of xenograft tumors orthotopically in the primary colon and generated spontaneous liver and/or lung metastasis. This model system effectively reproduces CRC as observed in human patients and provides detailed information about signaling networks involved in metastatic dissemination [15, 17, 18]. We compared the primary and liver metastatic tissues using microarray analysis and has identified gene signatures similar to the recent report on the comprehensive molecular characterization of CRC from The Cancer Genome Atlas Network [19]. Furthermore, we compared the cell proliferative capabilities of the GEO orthotopic mouse primary and metastatic liver tumors with patient's CRC tumors and observed similarity in their proliferative patterns. Therefore, our development of an orthotopic metastatic mouse model system of CRC might be utilized as a powerful tool to study late stages of the metastatic cascade that involves colonization of cancer cells to distant organ sites. 

## 2. Materials and Methods

### 2.1. Cell Culture

GEO cells were isolated from a primary tissue culture of a human colon carcinoma as described by Brattain et al. [20]. The GEO cells were adapted to grow in serum-free medium [15, 16, 21] consisting of McCoy's 5A medium (Sigma) supplemented with amino acids, pyruvate, and antibiotics (designated SM) containing the growth factors transferrin (4 *μ*g/mL; Sigma), insulin (20 *μ*g/mL; Sigma) and EGF (5 ng/mL; Collaborative Research). 

### 2.2. Green Fluorescence Protein (GFP) Transfection

Packaging cells, 293 GP (Clontech, Mountain View, CA, USA), were cotransfected with a plasmid encoding VSVG envelope protein and a retroviral vector encoding GFP and the G418 resistance gene using FuGene (Invitrogen, Carlsbad, CA, USA). The viruses were collected 48 h later and used to infect GEO cells. After 48 h, the infected GEO cells were selected by treatment with G418 for 5 days. This resulted in a stable transfection of GEO cells with GFP reporter. 

### 2.3. Orthotopic Implantation

All experiments involving animals were approved by the Institutional Animal Care and Use Committee. The orthotopic implantation methodology has been described in detail in earlier reports from our laboratory [15, 17, 22, 23]. GEO CRC cells were transfected with green fluorescence protein (GFP). Exponentially growing GFP-labeled GEO cells (approximately 7 million cells/mL) were inoculated subcutaneously onto the dorsal surfaces of separate Balb/c nude male mice. Once xenografts were established, they were excised and minced into 1-2 mm^3^ pieces. Orthotopic implantation procedure was performed using two of these pieces into other Balb/c nude mice. For operative procedures, animals were first anesthetized with isoflurane inhalation. A 1-cm laparotomy was performed and both the caecum and ascending colon were exteriorized. Using X7 magnification and microsurgical techniques, serosal layer was abraded by scraping in two locations of colon, 1 cm apart. The 1-2 mm^3^ pieces of xenograft were subserosally implanted using an 8-0 nylon suture at two areas of the abraded serosa. The bowel was then returned to the peritoneal cavity and the abdomen was closed with absorbable 5-0 vicryl suture and skin with 5-0 proline suture. This was followed with weekly GFP fluorescence imaging for up to 5 weeks after anaesthetizing animals with a 1 : 1 mixture of ketamine (10 mg/mL) and xylazine (1 mg/mL) by intraperitoneal injection (0.01 mL/mg). Excitation of GFP was captured in the light box illuminated by fiberoptic lighting at 470 nm (Illumatool BLS; Lightools Research, Encinitas, CA, USA) using a Retiga EXi color CCD camera (QImaging, Burnaby, BC, Canada). High-resolution images were captured directly using an MS Windows-based PC that facilitated identification of primary and metastatic diseases by direct near real-time visualization of fluorescence in live animals. Thirty-five days after implantation, animals were euthanized. Organs were explanted, imaged, and immediately placed in 10% neutral buffered formalin fixative for 12 to 24 h. Tissues were then processed and embedded in paraffin as blocks. 

### 2.4. Hematoxylin and Eosin and Ki67 Staining

Slides were cut from paraffin-embedded blocks using a microtome and stained with hematoxylin and eosin and Ki67 IHC using previously established protocols [21]. Serial sections were cut to complement the hematoxylin and eosin sections and were stained with IgG_1_ rabbit polyclonal antibody for Ki67 (Dako Corporation). Ki67 is a nonhistone nuclear antigen present in late G_1_, G_2_, and S phases of the cell cycle but not in G_0_. The optimal dilution of 1 : 20 dilution was used, and staining was performed following manufacturer's protocol. The proliferation rate was determined semiquantitatively by counting the number of positively stained proliferative cells per 75-*μ*m² field at 10x magnification. Approximately 1000 total cells were counted, and the percentages of positively stained cells were calculated. Human paraffin-embedded specimens of both the CRC primary tumor and the corresponding liver metastasis were obtained from University of Nebraska Medical Center Tissue Science Core and approved by Institutional Revenue Board. 

### 2.5. Bioinformatics Analysis

Heat maps and bioinformatics analysis were performed by the UNMC bioinformatics core facility using the Oncomine and Ingenuity Pathway Analysis (IPA) software tools. 

### 2.6. Statistical Analysis

GraphPad software (San Diego, CA, USA) was utilized for statistical analysis. All the experiments were repeated three times independently to determine consistency in the results. The results were expressed as mean ± SE for three replicates for each case.

## 3. Results

### 3.1. Orthotopic Metastatic Mouse Model of Colorectal Cancer Metastasis

Metastasis to the distant organs is the most common cause of cancer-related death in CRC, and understanding the molecular mechanisms of metastasis is critical to the development of effective therapies against metastasis for the improvement of patient survival with distant metastasis. A persistent issue with cancer research is the lack of *in vivo* mouse models that can effectively recapitulate the multistep metastatic process as observed in human patients [24]. Subcutaneous tumors generated by intraperitoneal injections of cancer cells generally grow faster and do not recapitulate the slower doubling times observed with most human solid tumors [24]. This potentially makes ectopic (subcutaneous) tumors more susceptible to chemotherapeutic agents targeting dividing cells. Furthermore, subcutaneous tumors do not metastasize due to tissue barrier [18]. Recent advancements in the generation of orthotopic xenograft transplantation models have greatly enabled us to study the various steps of the metastatic cascade with more efficiency and accuracy compared to ectopic models [24–27]. Spontaneous metastasis models that utilize surgical resection of primary tumors to allow sufficient time for the metastatic cells to survive and successfully colonize at distant secondary organ sites are currently being evaluated [24, 28–31]. 

**Figure 1:**
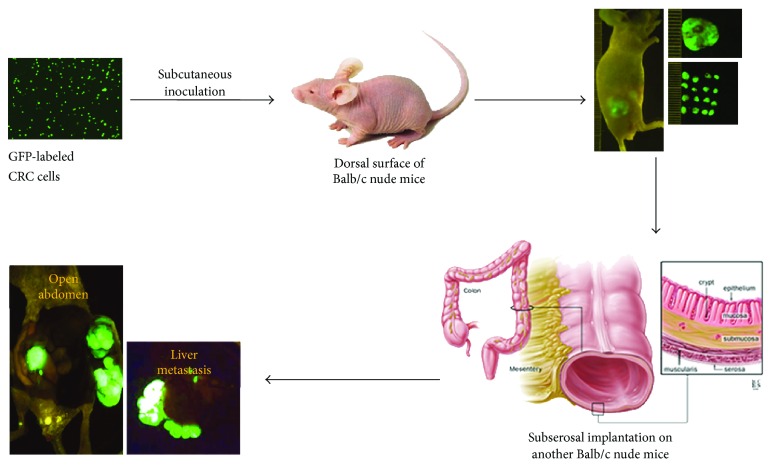
Orthotopic metastatic mouse model. The operative procedures are described in detail in Sections 2 and 3. Image for the different tissue layers of colon has been taken from the Johns Hopkins Medicine Gastroenterology & Hepatology website (http://www.hopkins-gi.org/).

**Figure 2:**
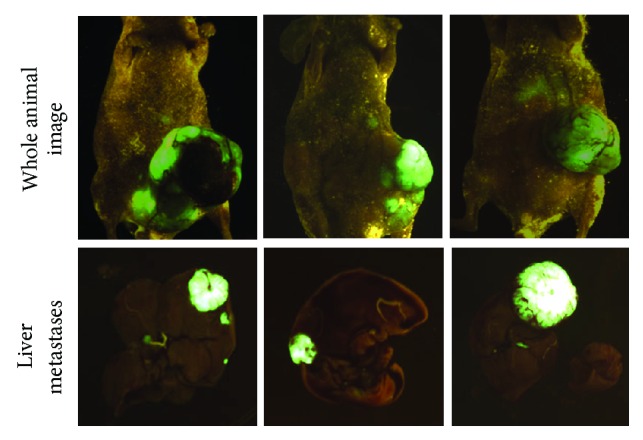
Development of GEO CRC primary tumors and liver metastasis using the orthotopic metastatic mouse model in Balb/c nude male mice.

**Figure 3:**
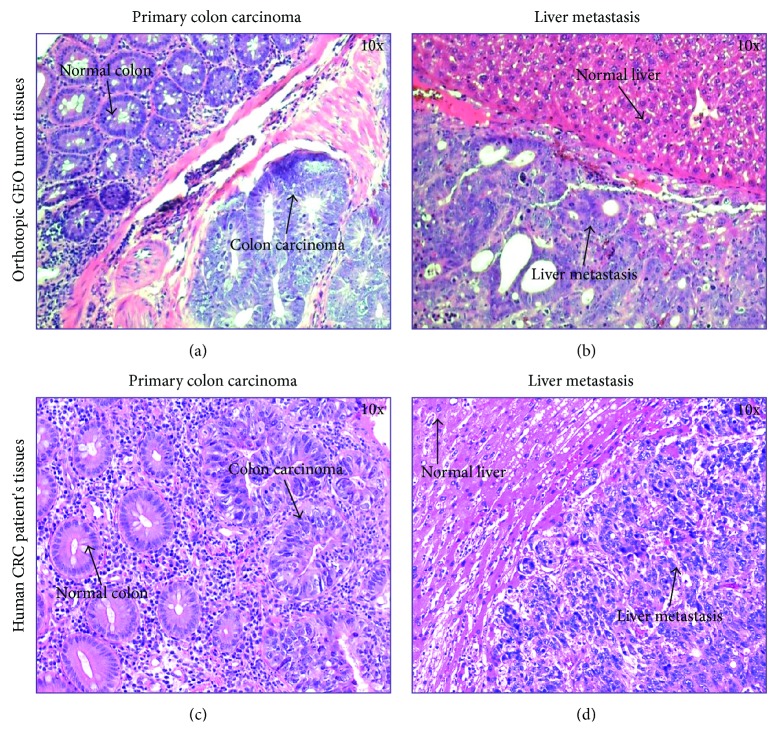
Eosin and hematoxylin staining of primary colonic and liver metastatic tissue sections from orthotopic GEO CRC tumors ((a), (b)) and human CRC patient's specimen ((c), (d)) showing normal and cancerous areas.

We have developed an orthotopic metastatic mouse model as shown in Figure 1. The detailed procedure has been described in Section 2. The metastatic pattern displayed in this model system reflects the nature of metastatic spread in human patients. Orthotopic metastatic mouse model allows for qualitative and quantitative reproducible metastatic colonization of liver and lungs, the main sites of metastasis in human CRC [15, 17, 18, 23]. Previously, we have shown that the GEO cells are highly metastatic [15]. The purpose of this study was to obtain data from the orthotopic metastatic mouse model using GEO human CRC cells that correlate with human CRC patient's specimen with the goal to recreate the sequential metastatic process observed in humans. We labeled GEO cells with GFP fluorescent tag for monitoring their primary tumor growth and metastasis *in vivo*. We observed the development of robust primary tumors by 4-5 weeks after implantation and liver metastasis in approximately 7-8 weeks from the time of initial implantation. The GFP-labeled images from closed abdomen and liver metastasis are shown in Figure 2. Paraffin-embedded blocks of the orthotopic GEO CRC primary tumors and liver metastases were cut into 4 *μ*m thick sections and stained using eosin and hematoxylin. Primary colon tumor and liver metastatic areas were observed as shown in Figure 3(a). Comparative eosin and hematoxylin staining was obtained from the human CRC patient's primary tumor and liver metastatic specimen as shown in Figure 3(b). 

### 3.2. Increased Cell Proliferation Is Associated with Liver Metastasis

**Figure 4:**
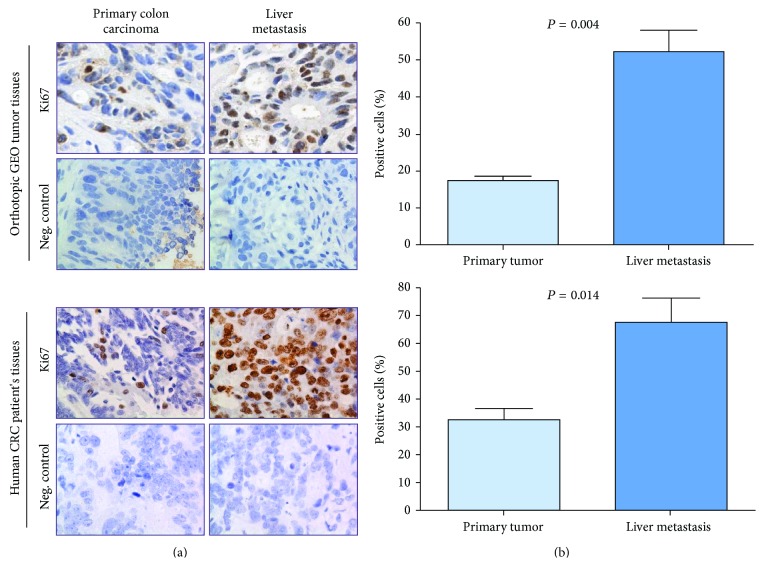
Increased cell proliferation in liver metastasis: Ki67 staining was performed on the primary colonic and liver metastatic tissue sections from orthotopic GEO tumors and human CRC patient's specimen (a) showing an increase in Ki67 staining of the liver metastatic tumors in both liver metastatic specimens. (b) Relative quantification was performed, followed by statistical analysis to quantify the increase in cell proliferation in liver metastatic tissues.

We analyzed the cell proliferation changes associated with metastasis in the orthotopic GEO primary tumors and liver metastatic tissues using the cell proliferation marker Ki67 staining method. Liver metastasis showed a statistically significant increase (*P* = 0.004) in cell proliferation compared with the CRC primary tumors (Figures 4(a) and 4(b) upper panels). Moreover, Ki67 staining for the human patient CRC primary tumor and the corresponding liver metastasis also showed a statistically significant increase (*P* = 0.014) in the cell proliferation of CRC liver metastasis in comparison with the primary tumor (Figures 4(a) and 4(b) lower panels). However, there was no significant change in the apoptotic cells measured by TUNEL assay in both the orthotopic implanted GEO tumors and human CRC specimens (data not shown). 

### 3.3. Microarray Analysis Profiling Gene Signatures Associated with GEO Primary Colon Carcinoma and Liver Metastasis

**Figure 5:**
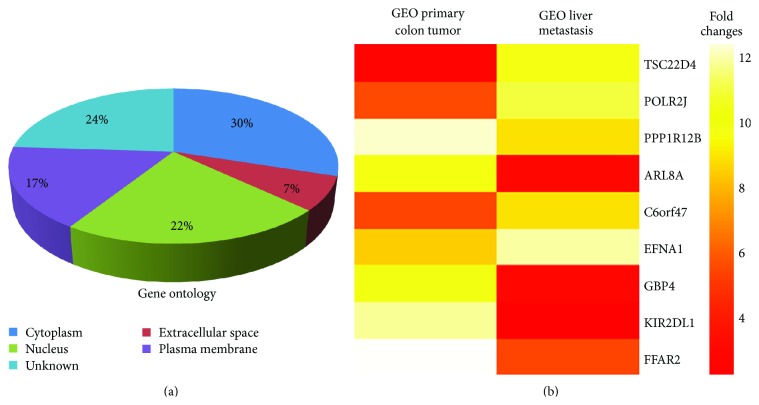
Bioinformatics analysis of GEO CRC primary colonic and liver metastatic tumors. (a) Gene ontology pie chart was prepared based on the heterogeneity of gene expression signature in the different subcellular locales. (b) Selective heat map dendrogram showing the markers for aggressiveness associated with CRC as recently reported in The Cancer Genome Atlas Network (TCGA) comprehensive analysis of CRC.

Next, we sought to determine the differences in gene expression between GEO primary colon carcinoma and liver metastasis tumor samples. Transcription profiles of the tumor samples were generated using the Affymetrix HGU133plus2.0 genechips and a heat map dendrogram was generated and ranked as shown in the Supplemental Figure 1 (see Supplementary Material available online at http://dx.doi.org/10.1155/2013/206875). Gene ontology (GO) analysis was performed to delineate the differences in gene expression signature between different cellular compartments. As shown in Figure 5(a), 30% of all genes were differentially regulated in the cytoplasm. There was a 17% difference in gene expression profiles in the plasma membrane, 22% in the nucleus, and 7% in the extracellular space. 

### 3.4. Identification of Markers for Aggressiveness of CRC

Recently, TCGA has comprehensively characterized the human CRC genome in 224 matched CRC patient tumors and their paired normal tissues for an integrative molecular insight into CRC and identified potential therapeutic targets [19]. The study reported the identification of several molecular signatures associated with tumor aggressiveness on the basis of tumor stage, lymph node status, metastasis, and angiogenesis at the time of surgery. The expression of many genes like TSC22D4, POLR2J, PPP1R, and C6ORF47 was dependent on the aggressiveness of the tumor. Interestingly, we observed similarity in the gene signatures associated with the aggressiveness of human CRC in the microarray data obtained by comparing GEO primary and liver metastatic tumors (Figure 5(b)). The genes which were upregulated in GEO liver metastasis similar to the TCGA study were TSC22D4, POLR2J, PPP1R, and C6ORF47. These genes have been implicated as markers of a more aggressive disease. In contrast, ARL8A, EFNA1, GBP4, KIR2DL1, and FFAR2 genes were downregulated in the GEO liver metastasis compared to primary tumors that had been described as markers of less aggressiveness. 

### 3.5. Expression of TSC22 Gene Family in CRC

**Figure 6:**
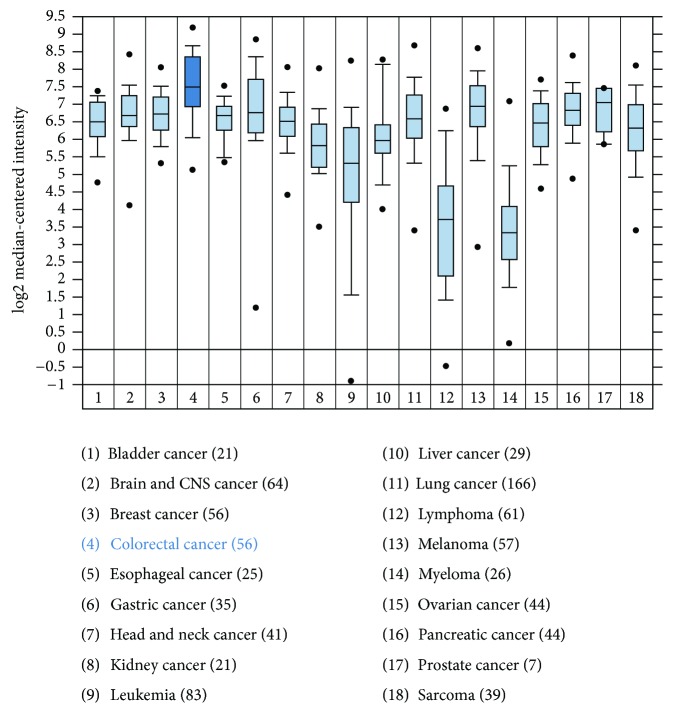
High TSC22D1 gene expression in CRC cell lines. The data is extracted from the deposited gene sets from Barretina and colleagues [36] and analyzed by Oncomine. 56 CRC cell lines were analyzed and that showed an overall 7.5-fold increase in TSC22D1 gene expression.

**Figure 7:**
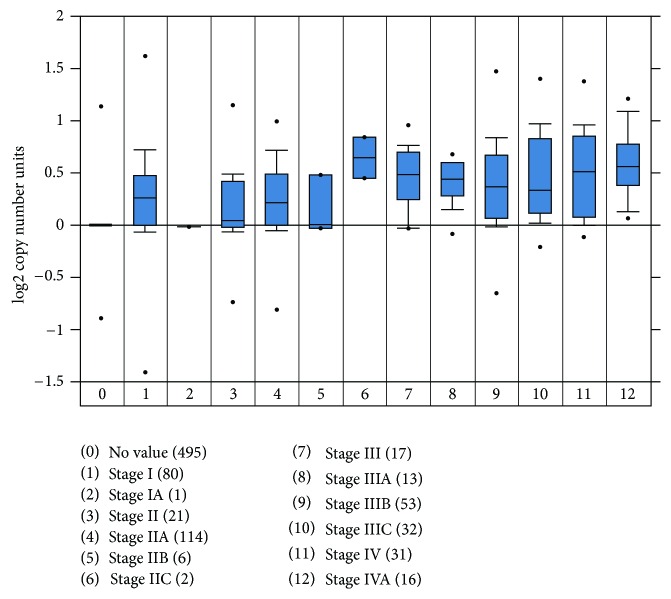
Increased copy number of TSC22D1 is associated with CRC progression.

The transforming growth factor *β* stimulated clone 22 (TSC22) domain family consists of 4 members (namely, TSC22D1–D4). It was first isolated from mouse osteoblast cells in response to TGF*β*. The functions of these genes are relatively unknown. While one study has reported TSC22D1 as a putative tumor suppressor [32, 33], another study has demonstrated antiapoptotic functions associated with TSC22D1 [34, 35]. TSC22 gene information was extracted from deposited gene expression data sets from Barretina et al. [36] using Oncomine analysis. TSC22D1 showed a 7.5 fold upregulation in colorectal cancer cell lines (average log2 median-centered intensity obtained from 56 cell lines) as shown in Figure 6. TSC22D1 mRNA expression was also high in other types of cancer. According to the gene expression data obtained from the TCGA database extracted and analyzed by Oncomine, TSC22D1 DNA copy number correlated with increased progression. As shown in Figure 7, increased expression of TSC22D1 was observed in stages IV and IVA compared to the earlier stages of CRC. Similar TCGA data was obtained for TSC22D4 using Oncomine (data not shown). However, no functional studies have been reported yet on the role of TSC22 domain family members in cancer progression and metastasis. 

## 4. Discussion

Metastases are largely responsible for cancer deaths in solid tumors due the lack of effective therapies against disseminated disease. Thus, there is an urgent need to fill this gap in cancer therapy. There are several tumor models available at present for preclinical studies for testing new therapies. However, these model systems have considerable shortcomings as recently reviewed in detail by Francia et al. [24]. Studies from the Kerbel laboratory [24] have indicated the importance of orthotopic transplantation model as pivotal to the recapitulation of the muti-step metastatic cascade. We have developed an orthotopic metastatic mouse model to study *in vivo* CRC metastasis. This model provides a powerful tool for the molecular and cellular characterization of the multistep metastatic cascade leading to the colonization of disseminated tumor cells to distant organ sites. As such, this model allows for reproducible quantitative analysis of metastases to the liver and lungs. The sequence of metastatic events observed in this model reflects the nature of metastasis in human CRC patients in that the liver and/or lung metastasis appears after the growth of primary tumors. The orthotopic metastatic mouse also serves as an important tool for preclinical drug evaluation. This model is further strengthened by its ability to assess *in vivo* fluorescent GFP imaging in real time for the assessment of tumor burden and metastatic deposition. 

In this study, we utilized the IGF1R-dependent GEO human CRC cell line that was isolated from a CRC patient's primary colonic carcinoma. Previously, we have shown that the GEO cell line is highly metastatic forming liver metastatic deposits within 7-8 weeks using the orthotopic metastatic mouse model [15]. Furthermore, we also demonstrated that GEO cells lack TGF*β* receptors due to epigenetic silencing [21, 37]. Restoration of TGF*β* type I receptor (TGF*β*R1) lead to a reduction in metastatic capability and activation of a tumor suppression pathway mediated through the TGF*β*/PKA signaling [15, 21]. This study compared the clinical relevance of the orthotopic metastatic mouse model to recapitulate and study the human CRC by comparing the cell proliferation changes associated with liver metastasis using colonic and metastatic tissues obtained from GEO tumors and human CRC patient's primary and metastatic tumors. We observed that liver metastatic tissues had a higher rate of Ki67 staining which is indicative of increased cell proliferation (Figure 4). Human CRC stage IV primary tumors and their corresponding liver metastasis were obtained as indicated in Section 2.5 for comparison with the orthotopic GEO tumors to analyze the difference in cell survival and proliferative capabilities. We obtained similar results in the human CRC specimen showing an increase in cell's aberrant proliferative capabilities in liver metastasis compared to primary colonic tumors (Figure 4). 

The Cancer Genome Atlas (TCGA) has recently comprehensively profiled the somatic changes associated with human CRC using a cohort of 276 patient samples [19]. The TCGA study analyzed exome sequence, DNA copy number, promoter methylation, and messenger RNA and microRNA expression. One of the common focal amplification observed in the cohort was that of insulin-like growth factor 2 (IGF2) which is part of the 11p15.5 chromosomal amplification. Furthermore, about 15% of tumors without IGF2 amplification showed about 100-fold higher expression of IGF2. It was revealed by the MEMo method [38] that systematically searches for mutually exclusive genomic events that IGF2 overexpression is correlated to the genomic events involved in PI3K activation suggesting that the IGF1R-IGF2-PI3K signaling might be a potential therapeutic target in CRC [19]. Previously, we have demonstrated that human GEO cells have a higher expression of IGF2 and are dependent on the IGF1R signaling for growth and survival [16]. Comparison of the primary colonic tumors and liver metastatic deposits showed significant heterogeneity in their gene expression (Supplemental Figure 1). We compared the novel gene signatures associated with aggressiveness to the TGCA report and observed an overlap of several of those genes, a subset of which is shown in Figure 5(b). We further characterized the TSC22 gene that has been indicated as a marker of tumor aggressiveness using Oncomine analysis. TSC22 is a TGF*β* inducible gene that is upregulated in several cancer types as shown in Figure 6. Interestingly, higher DNA copy number of TSC22D1 was observed in stages IV/IVA indicating towards a potential role of TSC22 domain family in enabling dissemination of late-stage carcinoma cells (Figure 7). 

## 5. Conclusion

We demonstrated the utilization of orthotopic metastatic mouse model to study CRC metastasis and showed reproducibility of gene signatures associated with aggressiveness of CRC. The GEO orthotopic metastasis model also showed an increase in cell proliferation associated with liver metastasis compared to primary colon carcinoma by Ki67 staining that was observed to compliment results obtained from human CRC patient's specimens. We demonstrated the importance of using the xenograft transplantation model in mouse to study metastatic colonization that appropriately reflects human CRC disease. This model can provide valuable information about effectiveness of new therapeutic strategies. Additionally, the orthotopic metastatic mouse model can be effectively utilized for the identification of genes that are playing a critical role in the development and/or maintenance of established CRC metastases. Finally, such studies would provide a basis for the development of novel strategies aimed at molecular targets with demonstrated potential for being relevant targets that will directly affect the metastatic disease. The development of new strategies for treatment of metastases is vital in the war against cancer because disseminated disease is by far one of the leading causes of cancer-related deaths, and there are very few drugs that have a significant effect on survival in these patients.

## Supplementary Material

Supplemental Figure: Microarray Analysis Profiling Gene Signatures Associated with GEO Primary Colon Carcinoma and Liver Metastasis. Fresh snap frozen tumor tissue sections were utilized for RNA isolation followed by microarray analysis.
