# Supplementary material for: Establishment and Validation of an Orthotopic Metastatic Mouse Model of Colorectal Cancer
Source: ISRN Hepatol. 2013 Apr 21;2013:206875. doi: 10.1155/2013/206875 (PMC4907346; doi:10.1155/2013/206875)
Supplement: Supplementary file 1 — Supplemental Figure: Microarray Analysis Profiling Gene Signatures Associated with GEO Primary Colon Carcinoma and Liver Metastasis. Fresh snap frozen tumor tissue sections were utilized for RNA isolation followed by microarray analysis. [file 206875.f1.pdf]

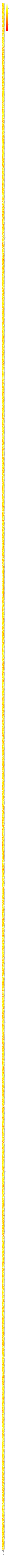
10
5
0
−5
−10
